# A “Self-Milieux” perspective on help-seeking: examining the impact of a person’s sociocultural background on help-seeking in people with untreated depressive symptoms

**DOI:** 10.1007/s00127-024-02720-3

**Published:** 2024-08-04

**Authors:** Thomas McLaren, Lina-Jolien Peter, Samuel Tomczyk, Holger Muehlan, Georg Schomerus, Silke Schmidt

**Affiliations:** 1 Department of Health and Prevention, Institute of Psychology, University of Greifswald, Robert-Blum Str. 13, 17489 Greifswald, Germany; 2 Department of Psychiatry and Psychotherapy, Medical Faculty, University Leipzig, Leipzig, Germany; 3 Division of Medical Psychology, Medical Department, Health & Medical University Erfurt, Erfurt, Germany

**Keywords:** Self-Milieux, Socioeconomic status, Self-construal theory, Sociocultural background, Cluster analysis, Help-seeking behaviour

## Abstract

**Background:**

Mental illness is a global concern and the leading cause of years lived with disability. Research on help-seeking behaviour has focused on individual factors, but there is still much unexplained variance. Suggesting complex interactions between determinants of human behaviour a new framework called *Self-Milieux* is proposed to represent a person’s sociocultural background. The article introduces a statistical approach to determine *Self-Milieux* and exemplarily examines its predictive validity for health-related research.

**Methods:**

*Self-Milieux* are determined through a two-stage clustering method based on the determinants socioeconomic status and self-construal profile. Descriptive analyses are used to compare *Self-Milieux* characteristics. Hierarchical binary logistic regression models test the association between *Self-Milieux* and help-seeking behaviour, while controlling for socioeconomic status as an established predictor.

**Results:**

The sample size was *N* = 1535 (*M*_*age*_ = 43.17 and 64.89% female participants). Average depression severity was *M* = 12.22, indicating mild to moderate symptoms. Six *Self-Milieux* were determined and named. Participants from *privileged* (*aOR* = 0.38) and *self-sufficient* (*aOR* = 0.37) *milieux* were less likely to seek help from a general practitioner than those from the *entitled milieu*. Participants from *privileged* (*aOR* = 0.30), *collaborators* (*aOR* = 0.50), *disadvantaged* (*aOR* = 0.33), and *self-sufficient* (*aOR* = 0.21) *milieux* were less likely to seek help from family members than those from the *entitled* and *family-bound milieux*.

**Discussion:**

The study’s strengths and limitations, as well as the cluster methodology, are discussed. The comparative results for the six *Self-Milieux* are interpreted based on current research. For example, participants from some *milieux* follow a help-seeking process proposed in previous research, while participants from other *milieux* seem to show a different process, one that ends in informal help-seeking.

**Supplementary Information:**

The online version contains supplementary material available at 10.1007/s00127-024-02720-3.

## Introduction

The prevalence of mental illness is a global concern, even in modern countries with formalised mental health care systems [1, 2]. Despite the availability of professional help, people often do not seek it or only after significant delay [3]. In Germany, this issue of non-help-seeking remains even in regions with high health care density [4, 5]. Understanding the reasons for this issue is crucial because mental illness contributes significantly to the global burden of disease and has substantial individual-level consequences [6, 7]. For example, mental illness is the leading cause of years lived with disability among all disease groups and untreated mental illness has significant economic costs for societies [8].

To address help-seeking behaviour in individuals with mental illness, psychological research has focused on various individual factors such as age, gender, personal attitudes, and influences of stigma [9, 10, 11, 12, 13]. Despite these efforts, there is still considerable unexplained variance in help-seeking behaviour of individuals, suggesting that complex interactions between determinants of human behaviour on individual and socio-contextual levels remain unclear [14, 15]. Consequently, there is a need to adopt a more interdisciplinary approach in health-related research and examine the individual as an agent of their own life who is also socialised into certain social and cultural constraints [16, 17, 18]. It has been suggested that a broader perspective on social and cultural determinants might help understand the help-seeking process beyond traditional health behaviour modelling approaches [18, 19].

**Fig. 1:**
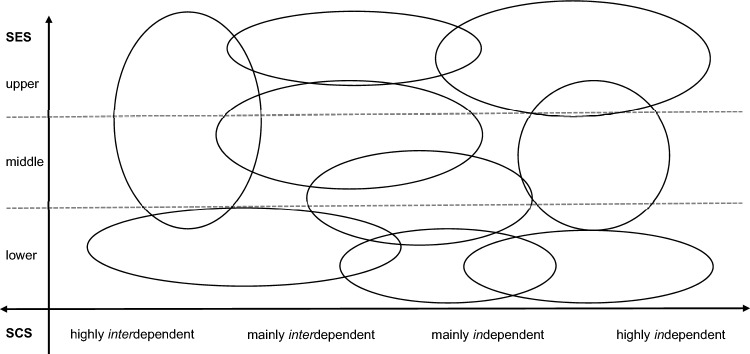
The *Self-Milieux* concept. The vertical dimension displays three socioeconomic status groups. The horizontal dimension displays a persons’ self-construal that ranges from highly *inter*dependent to highly *in*dependent. The ovals represent arbitrarily placed *milieux*

Therefore, a novel framework titled *Self-Milieux* is proposed that conceptualises and interprets human behaviour within a sociological framework, using the social milieu paradigm [20, 21]. This paradigm relates two orthogonal dimensions to each other, describing a person’s social status differentiated by subjective qualities along the horizontal dimension [21]. When social milieus are studied it is assumed that people within a milieu share ethical and aesthetic attitudes due to similar mentalities [21]. The novel framework is aligned with this paradigm, however, in comparison to the social milieu framework, the *Self-Milieux* incorporate the concept of self-construal, which was developed in cross-cultural comparative psychology [22, 23, 24]. The *Self-Construal Theory* [22] proposes that individuals can have either an *in*dependent or *inter*dependent self-construal, depending on cultural and social contexts [24]. Individuals with a more *in*dependent self-construal regard themselves as separate from their contexts [25] and consistent across situations [26], whereas, individuals with a more *inter*dependent self-construal perceive themselves to be more role- and context-bound [27, 28]. This theory is relevant for understanding human emotion, motivation, and behaviour [23]. By incorporating the *Self-Construal Theory* within the social milieu paradigm, both the individual as well as the social level are recognised as relevant determinants within the *Self-Milieux* framework. Methodologically, the *Self-Milieux* are determined within a two-by-two coordinate system, similar to the social milieu concepts. The vertical dimension is defined by the *socioeconomic status* (SES) of the individual, and the horizontal dimension is defined by the *self-construal profile* (SCS) of the individual. This framework accounts for sociocultural factors that influence individual behaviour, which could provide a more comprehensive understanding of health-related help-seeking [19]. The *Self-Milieux* are visualised in Fig. 1. Individuals in the same *milieu* are assumed to have similar concepts of self, influenced by their respective socio-economic background and culturally related self-construal profile.

The determinants to conceptualise *Self-Milieux* are chosen for two main reasons. Firstly, international research has considered both SES and self-construal concerning a multitude of psychological and sociological phenomena [e.g., 23, 24, 29, 30]. Secondly, in reciprocal processes of socialisation the regional SES and the culturally influenced understanding of the self has lasting effects on individuals influencing people across different personal and social domains of living [e.g., 17, 30, 31]. Furthermore, the *Self-Milieux* are proposed to be relevant in the context of health related research, because both SES and SCS have been associated with health-related behaviour and health inequality, which will be elaborated in the following sections [e.g., 32, 33, 34, 35, 36, 37].

The role of SES has been studied comprehensively in the context of health-related inequality [e.g., 34]. Phelan et al. [38] have even defined SES as a ‘fundamental cause’ of health-related inequality in mortality. The SES markers education, occupation, and income have been shown to have a significant impact on health-related opportunities, with individuals from lower SES backgrounds experiencing shorter and more health-burdened lives [39]. Additionally, regional SES has also been found to impact health outcomes [40]. Lower SES is associated with poorer health evaluation, symptom expression, and overall quality of life [41, 42]. The importance of SES in the context of health-related research is undeniable. However, it is necessary to allow for horizontal differentiation to account for inconsistencies within status groups [41]. Furthermore, the differentiation allows the influence of cultural factors to be explicitly addressed in empirical investigations.

To provide a comprehensive concept, the incorporation of self-construal as a subjective factor is proposed. It is argued that individuals are not only socialised within and into a certain SES [17, 43, 44] but, also, socialised within and into an understanding of self in relation to other [23, 24]. Self-construal is a socio-culturally influenced, overarching self-schemata that organises and regulates one’s experiences and actions, and influences intra- and inter-psychological functions, including cognitive processes, emotions, and intra-/inter-group relations [22, 23, 24, 26, 27, 28, 31, 45, 46]. More importantly for this study, a person’s self-construal has been shown to have relevant implications for health-related behaviour in different contexts, including the subjective experience of illness and protective factors against depression [e.g., 23, 47, 48, 49].

Considering the above, the primary aim of this article is two-fold. Firstly, to introduce a statistical approach to methodologically determine the concept of *Self-Milieux*, which represents a person’s sociocultural background instilled through socialisation. Secondly, to exemplarily show the predictive validity of the concept for health-related research, the *Self-Milieux* are examined regarding the help-seeking process of individuals with depressive symptoms. The help-seeking process involves *self-identifying the complaints as symptoms of mental illness*, forming an *intention to seek help*, and engaging in *help-seeking behaviour* [50], [51], [52].

## Methods

The data for this study is taken from a project funded by the *German Research Foundation* (DFG), which looks at ways in which professional health care utilisation can be improved. The overall project has a study protocol [53] and was preregistered in the *German Clinical Trial Register* (https://drks.de/search/de/trial/DRKS00023557). The analyses done in this study are in addition to the research questions reported in the study protocol. Data was collected via an online panel between January and September 2021.

### Sample

Participant recruitment was conducted with a German online-panel. Prospective participants (*N* = 10,348) were screened and included if they were currently not receiving health care treatment and had at least mild depressive symptoms [referring to a PHQ-9 sum score of 8 or higher; 54]. Altogether, a convenience sample of *N* = 1867 participants was included in the study. Before data analysis participants were excluded if they completed the survey within less than half the median study duration [55] or had apparent monotone answer profiles (*n* = 116), due to disparate gender data between the two study points (*n* = 15), and because they were of diverse gender (*n* = 9), leaving a sample size of *N* = 1727. After calculating the relevant SES indices (see further below), *n* = 192 were excluded from further analyses due to missing data concerning the variable “net household income”. Therefore, *N* = 1535 participants are included in the statistical analysis.

### Measures

To measure the *socioeconomic status*, the *SES-Index* is determined using sociodemographic data, i.e., level of acquired schooling and education, type of vocation, and net household income level. The method for this is described in Lampert et al. [56]. A three-dimensional index is calculated on the basis of the meritocratic indices education, vocation, and income. The separate index values range from 1 = “low status” to 6 = “high status” and are summed up. The *SES-Index* values range from 3 = “low socioeconomic status” to 18 = “high socioeconomic status”. The wordings for the sociodemographic variables and exact calculations for the indices are reported in the supplementary material (Table S1).

To determine the participants status group, five quintiles are calculated for the summed *SES-Index*. The first quintile represents the “upper”-, the next three represent the “middle”-, and the fifth quintile represents the “lower”-status group. Two benefits arise from calculating the status in this way: first, “the distribution-based differentiation of the status groups underlines that the socioeconomic status is a measure of relative social inequality” and second, “it is ensured that there are no distortions in correlation-based analyses due to extreme cell occupations in the categories” [translated from 56]. The three meritocratic indices are separately used in the cluster analysis methodology to determine the *Self-Milieux*. When comparing the *milieux* the three status groups will be used.

To measure the participants *self-construal*, 24 items from four *Self-Construal Scale* dimensions are used, i.e., the dimensions representing the domains of personal and social functioning “making decisions”, “looking after oneself”, “communicating with others”, and “dealing with conflicting interests” [31]. Each dimension is assessed with six items, three reflecting a more *in*dependent and three a more *inter*dependent self-construal, respectively. Response options are 1 = “does not describe me at all” to 5 = “describes me exactly”. In the supplementary material the *in*dependent vs. *inter*dependent expressions of the dimensions as well as the items are reported (Table S2). Internal consistency across all items is very good (α = 0.82).

To calculate the separate dimension scores, first, the mean score is calculated across all items and then the acquiescence bias is corrected for by subtracting the mean score from the individual item [as suggested; 57]. The measure therefore reflects the individuals relative endorsement of each item in the context of the respective measurement occasion [31]. Second, the items measuring the *inter*dependent self-construal are reversed. Third, the mean across the items within a dimension is calculated.

To determine the overall self-construal identification of the participants the mean across all four dimensions is calculated. Individuals with mean scores between − 1.5 and 0 will be classified as “mainly *inter*dependent” and individuals with scores between 0 and + 1.5 will be classified as “mainly *in*dependent”. The four dimensions are separately used in the cluster analysis methodology to determine the *Self-Milieux*. When comparing the *milieux* the two classifications will be used.

*Age* and *gender* (“female” and “male”) were assessed together with the other sociodemographic variables used to determine the *socioeconomic status*.

*Depression severity* was assessed using the *PHQ-9* questionnaire [58]. Participants reported how often they had complaints over the past two weeks (e.g., little interest or pleasure in doing things). Response options were 0 = “not at all”, 1 = “several days”, 2 = “more than half the days”, 3 = “nearly every day”. Sum scores (range = 0 to 27) are calculated and higher scores indicate greater severity of reported depressive symptoms. Internal consistency is acceptable (α = 0.70).

*Self-identifying complaints as symptoms of mental illness* was assessed using the *SELF-I* questionnaire [51]. Participants appraised their current problems (e.g., my present problems could be the first signs of a mental disorder) on a 5-point Likert scale from 1 = “don’t agree at all” to 5 = “agree completely”. Scores for the items 2, 4, and 5 are reversed before the mean score is calculated. Internal consistency is very good (α = 0.88).

*Help-seeking intention* was assessed using an adapted 15-item list [59]. Answers could be given on a 7-point Likert scale from 0 = “extremely unlikely” to 6 = “extremely likely”. We will conduct analyses for intention to seek help from a *general practitioner* (GP), a *mental health professional* (MHP; i.e., psychologist, psychotherapist, or psychiatrist), a *social helper* (i.e., social worker, counselling centre, teacher), a *spiritual leader* (i.e., priest or soothsayer), as well as informal instances, i.e., a *friend* or a *family member*. Maximum scores across the (grouped) items are taken to receive indicators for a participant’s intention to seek help. Other sources of help-seeking are not included in the analyses, e.g., police, colleague, or neurologist.

*Current help-seeking behaviour* was assessed using the same list to assess intention. The participants could state whether they had sought help for their psychological complaints during the past six months using a binary format (0 = “no” and 1 = “yes”). The same groups as described above for assessing intention are used in these analyses.

*Previous help-seeking experience* was assessed by asking participants following question: “have you ever had treatment for mental illness in your life?” Answers could be given in a binary format for different treatment types. In this study responses concerning the answer categories “medical treatment”, “psychotherapy”, “art-, music- and/or sport-therapy”, “self-help groups”, and “coaching and counselling” are considered and collapsed into one variable (0 = “has no experience” and 1 = “has experience”).

### Methodologically determining the *Self-Milieux*

In accordance with the findings of Stephens et al. [24], it is proposed that cultural factors influence self-construal. Furthermore, it is argued that the manner in which this self-construal is formed in the context of a specific sociocultural environment (i.e., social class) is relevant. To put it another way, a culture that encourages independence or interdependece will foster it in different ways depending on the social background of the individual. To determine the *Self-Milieux* representing the sociocultural background of an individual, a two-stage clustering analysis method is executed [60]. The SES indices education, vocation, and net household income, as well as the four self-construal dimensions developed by Vignoles et al. [31] are used as analysis input. Together, the seven dimensions map out different sociocultural backgrounds that go beyond the original constitutional variables in a Gestalt idea. In the first stage, all cluster solutions using a hierarchical method are determined and cluster centroids for the chosen solution are calculated. In the second stage, the hierarchically determined centroids are used as initial seed points in a non-hierarchical method. The advantage of the two-stage method is that this allows for correction of possibly improper cluster membership assignments due to the issue that hierarchical cluster analysis methods strictly nest the solutions, restricting cluster membership re-assignment. The partitioning method “unnests the hierarchy and allows for all data units to be assigned to the nearest cluster centroid” while still using the hierarchically found centroids as seed points [60].

The hierarchical method used is described by Ward [61]. The non-hierarchical method used is described by MacQueen [62]. The analyses are all done using the statistics software *R version 4.1.0*. [63]. To implement the hierarchical cluster method, the *agnes* {cluster} function [64] is conducted and doubled checked using the *hclust* {stats} function [63]. Dendrograms are used to choose a sufficient cluster solution. The centroids of the chosen cluster solution are calculated and used as the initial seed points in the function *kmeans* {stats}. These cluster memberships are then used to define the cluster membership, i.e., the *Self-Milieux*. Cohens Kappa [65] is calculated to compare the cluster solutions between the hierarchical and non-hierarchical solutions. The z-standardised variables for the three separate SES indices and the four SCS dimensions are used as analysis input variables. All R-code specifications to determine the *Self-Milieux* are supplied in the supplementary material (Table S3).

Concerning the a priori determined statistical power, Dalmaijer et al. [66] conclude that even relatively small samples (*n* = 20 per subgroup) are sufficient, and recommend *n* = 20 to 30 per expected subgroup.

### Statistical and power analyses

Descriptive analyses to investigate the characteristics of the *Self-Milieux* are conducted with the demographic variables *age* and *gender*, with *depression severity*, as well as with the help-seeking variables *self-identifying* complaints as symptoms of mental illness, help-seeking *intention* and *behaviour*, and *previous treatment experience*. Univariate ANOVAs with Bonferroni-adjusted post hoc comparisons or Chi-square tests with adjusted residuals (*AR*) for each cell are used to compare the groups. *AR* bigger than 2 or smaller than -2 are considered to be significant [67].

Four hierarchical binary logistic regression models are performed to test for the associations of *Self-Milieux* and help-seeking behaviour (criteria: *general practitioner*, *mental health professional*, *friends*, *family*). It is controlled for *age*, *gender*, *depression severity*, and *SES* as a means to analyse incremental validity (model 1, respectively). Reference *milieux* will be chosen depending on the descriptive analyses, i.e., the milieu in which participants show the most help-seeking behaviour (model 2, respectively). This way the robustness of the descriptive results are checked. Nagelkerke’s *Pseudo R*^*2*^ is reported [68].

The necessary sample size was calculated a priori using G*Power [69]. For the ANOVAs, *N* = 324 is sufficient (medium effect size, α = 0.05, 1−β = 0.95, six groups). For the logistic regression analyses, *N* = 988 is sufficient (OR = 1.3, Pr(Y = 1) H0 = 0.2, α = 0.05, 1−β = 0.95).

Cluster analysis to determine the *Self-Milieux* is conducted using the statistics software *R version 4.1.0* [63]. All other analyses are conducted using the statistics software *IBM SPSS* version *29*. Significance levels are set to *p* < 0.05.

## Results

### The *Self-Milieux* determined through the two-stage cluster analysis method

The cluster solution with six clusters is chosen as appropriate, based on the dendrograms of both the cluster analyses with a cut-off at Hight 25, as well as conceptual consistency and explanatory plausibility [see: 62].The cluster allocation is the same irrespective of the algorithm. We considered it plausible that six clusters could be discerned and described within the current study sample, with minimal overlap between the clusters. However, the method itself is exploratory and therefore inherently decisionistic. The *hclust* {stats} dendrogram is reported in the supplementary material (Fig. S4). The cluster centroids for the six clusters chosen as the best cluster solution can be found in the supplementary material (Table S5). These centroids are then used as the initial seed points for the partitioning method. After running the *kmeans* {stats} algorithm with the centroids as seed points, the participants are allocated to the nearest cluster. In the supplementary material (Table S6) the cluster allocations are shown. Cohens Kappa is moderate with Κ = 0.57 (95% CI 0.54; 0.59) when comparing the hierarchical with the non-hierarchical cluster solution.

Table 1 shows the different SES groups and SCS profile distributions in the six *Self-Milieux*. Additionally, the *milieux* are described according to the respective determinants with a creative label (see Table S5 in the supplementary material for the exact expressions along the seven dimensions). *Milieu 1*, “the privileged”, includes people from mainly upper SES, especially with high income, and mainly *in*dependent self-construal showing high levels of self-interest when dealing with conflicting interests. *Milieu 2*, “the collaborators”, includes people from mainly middle SES, especially higher vocational status, and high *inter*dependent self-construal. *Milieu 3*, “the entitled”, includes people from mainly middle SES and mainly *in*dependent self-construal, except for showing a tendency to depend on others when looking after oneself. *Milieu 4*, “the family bound”, includes people from both middle and lower SES and higher *inter*dependent self-construal. *Milieu 5*, “the disadvantaged”, includes people from both middle and lower SES and mainly independent self-construal. *Milieu 6*, “the self-sufficient”, includes people from mainly middle SES and more independent self-construal, except for showing a tendency to desire harmony when communicating with others.

**Table 1:** Distribution of socioeconomic status (upper, middle, and lower status) and self-construal profiles in the six *Self-Milieux*

**Self-Milieux**	**Socioeconomic status**			**Self-construal profile**		*n* in each milieu
	Upper	Middle	Lower	Mainly interdependent	Mainly independent	
1: “the privileged”	200 (70.18%)	85 (29.82%)	0	66 (23.16%)	219 (76.84%)	285
2: “the collaborators”	59 (28.37%)	149 (71.63%)	0	204 (98.08%)	4 (1.92%)	208
3: “the entitled”	25 (9.33%)	232 (86.57%)	11 (4.10%)	116 (43.28%)	152 (56.72%)	268
4: “the family bound”	0	114 (42.70%)	153 (57.30%)	257 (96.25%)	10 (3.75%)	267
5: “the disadvantaged”	0	118 (44.70%)	146 (55.30%)	2 (0.76%)	262 (99.24%)	264
6: “the self-sufficient”	38 (15.64%)	200 (82.30%)	5 (2.06%)	41 (16.87%)	202 (83.13%)	243
Σ	322 (21%)	898 (58.5%)	315 (20.5%)	686 (44.7%)	849 (55.3%)	135

Frequencies reported in n (%). Frequencies in the milieux consider the “n in each milieu” as the reference

### Sample characteristics & descriptive analyses

The final sample size consisted of *N* = 1535 at baseline and *N* = 1125 three to six months later. The sample mean age is 43.17 (*SD* = 15.42) years old with 64.89% of the participants identifying as female and an average depression severity of 12.22 (*SD* = 4.14). This indicates that participants mainly report mild to moderate symptoms of depression [54]. Most participants had the German equivalent of the “international baccalaureate” (51.7%), followed by an “average ten years of schooling” (34.7%) and “nine or less years of schooling” (12.9%). Concerning net household income, most participants reported “1000–2000€ net income” (36.1%), followed by “less than 1000€” (28.6%), “2000–3000€” (20.1%), and “more than 3000€” (9.8%). In Table 2 the sample characteristics including the results of the descriptive comparative analysis, both ANOVA/ANCOVA as well as Chi-square tests, are shown in which the *Self-Milieux* are compared with each other.

**Table 2:** Overall sample characteristics as well as descriptive analyses results comparing the milieux to each other. Statistical tests were either ANOVA/ANCOVA (continuous variables) or Chi-square test (categorical variables)

		**Self-Milieux**						**Statistical Tests**		
	**Total**	**Milieu 1**	**Milieu 2**	**Milieu 3**	**Milieu 4**	**Milieu 5**	**Milieu 6**	**test**	**η**^2^	**Sig.**
N (baseline)	1535	285	208	268	267	264	243			
**Age**^(x)^	43.17 (15.42)	43.51 (13.81)^a/b^	42.38 (13.89)^c^	43.79 (14.32)^d/e^	38.60 (17.40)^a/d/f^	41.64 (16.69)^g^	49.43 (13.80) ^b/c/e/f/ g^	F (5, 1529)= 14.001	0.044	< 0.001
								χ^2^ (5)= 46.700		< 0.001
**Gender**^(z)^										
female	996 (64.89 %)	150 (-4.8)*	147 (+1.9)	160 (-2.0)*	207 (+4.8)*	182 (+1.5)	150 (-1.1)			
male	539 (35.11%)	135 (+4.8)*	61 (-1.9)	108 (+2.0)*	60 (-4.8)*	82 (-1.5)	93 (+1.1)			
**PHQ-9**^(x)^	12.22 (4.14)	11.41 (3.74)^a/b/c^	12.75 (3.97)^a^	11.74 (4.09)^d^	12.78 (4.39)^b/d^	12.74 (4.51)^c^	12.05 (3.86)	F (5, 1529)= 5.603	0.018	< 0.001
**SELF-I**^(y)^	3.23 (0.88)	2.96 (0.83)^a/b/c^	3.50 (0.81)^a/d/e^	3.16 (0.82)^d^	3.34 (0.87)^b^	3.34 (0.92)^c^	3.15 (0.95)^e^	F (5, 1517)= 7.741	0.025	< 0.001
**Intention**^(y)^										
general practitioner	2.68 (2.01)	2.60 (1.89)^a^	2.73 (1.93)	3.16 (1.95)^a/b/c^	2.45 (2.03)^b^	2.67 (2.11)	2.49 (2.09)^c^	F (5, 1516)= 4.997	0.016	< 0.001
mental health professional	2.37 (2.08)	2.50 (1.99)^a^	2.45 (1.99)^b^	2.88 (2.08)^c/d/e^	2.34 (2.06)^c/f^	2.23 (2.16)^d^	1.77 (2.04)^a/b/e/f^	F (5, 1517)= 8.961	0.029	< 0.001
social helper	1.42 (1.79)	1.54 (1.78)^a^	1.14 (1.62)^b^	1.89 (1.92)^b/c/d/e^	1.42 (1.70)^c^	1.36 (1.85)^d^	1.05 (1.68)^a/e^	F (5, 1517)= 3.104	0.023	< 0.001
spiritual leader	0.55 (1.29)	0.78 (1.57)^a/b^	0.49 (1.17)	0.74 (1.52)^c/d^	0.37 (0.93)^a/c^	0.52 (1.29)	0.34 (1.02)^b/d^	F (5, 1517)= 6.540	0.021	< 0.001
friends	2.86 (2.22)	3.21 (2.10)^a^	2.70 (2.16)^b^	3.38 (2.14)^b/c/d/e^	2.88 (2.23)^c^	2.79 (2.30)^d^	2.09 (2.21)^a/e^	F (5, 1517)= 7.256	0.023	< 0.001
family	2.73 (2.16)	2.88 (2.10)^a/b/c^	2.75 (2.05)^d/e^	3.43 (2.08)^a/d/f/g/h^	2.84 (2.12)^f/i^	2.29 (2.26)^b/e/g/i^	2.09 (2.10)^c/h^	F (5, 1517)= 11.153	0.035	< 0.001
**Treatment experience**^(z)^	1476	281	204	256	260	249	226			
general practitioner	354 (23.98%)	51 (-2.5)*	54 (0.9)	59 (-0.4)	53 (-1.5)	80 (3.3)*	57 (0.5)	χ^2^ (5)= 17.159		0.004
mental health professional	581 (39.36%)	93 (-2.4)	82 (0.3)	100 (-0.1)	100 (-0.3)	122 (3.4)*	84 (-0.7)	χ^2^ (5)= 14.918		0.011
art/music/sport therapy	91 (6.17%)	8 (-2.6)*	10 (-0.8)	12 (-1.1)	20 (1.1)	29 (3.9)*	12 (-0.6)	χ^2^ (5)= 21.143		< 0.001
self-help group	72 (4.88%)	9 (-1.4)	7 (-1.0)	13 (0.2)	12 (-0.2)	20 (2.5)*	11 (0.0)	χ^2^ (5)= 8.019		0.155
coach/counselling	103 (6.98%)	21 (0.4)	14 (-0.1)	13 (-1.3)	14 (-1.1)	31 (3.7)*	10 (-1.6)	χ^2^ (5)= 16.305		0.006
N (follow-up)	1125	217	143	189	189	198	189			
**Current behaviour**^(y)^										
general practitioner	276 (24.53%)	43 (-1.8)	39 (0.8)	61 (2.7)*	43 (-0.6)	54 (1.0)	36 (-1.9)	χ^2^ (5)= 13.505		0.019
mental health professional	135 (12.00%)	19 (-1.6)	17 (0.0)	27 (1.1)	25 (0.6)	23 (-0.2)	24 (0.3)	χ^2^ (5)= 3.484		0.626
social helper	54 (4.80%)	10 (-0.1)	6 (-0.4)	11 (0.7)	10 (0.3)	12 (0.9)	5 (-1.5)	χ^2^ (5)= 3.270		0.658
spiritual leader	24 (2.13%)	10 (2.8)*	2 (-0.7)	3 (-0.6)	3 (-0.6)	3 (-0.7)	3 (-0.6)	χ^2^ (5)= 7.908		0.161
friends	426 (37.87%)	87 (0.8)	52 (-0.4)	74 (0.4)	83 (1.9)	82 (1.1)	48 (-3.9)*	χ^2^ (5)= 17.216		0.004
family	413 (36.71%)	73 (-1.0)	60 (1.4)	90 (3.4)*	90 (3.4)*	61 (-1.9)	39 (-5.0)*	χ^2^ (5)= 45.926		0.001

Superscript letters (a to i) highlight significant, Bonferroni-adjusted post-hoc results between the milieux concerning the same variable

Stars (*) highlight significant adjusted residuals (in brackets) between the milieux concerning the same variable. -/+ shows whether there is less/more than expected in the respective category

ANOVAs (x) & ANCOVAs (y), controlled for the variables age and depression severity, done with continuous variables; *M (SD)* reported

Chi-square (z) done with categorical variables; *N (AR)* reported unless specified as *N (%)*

For behaviour and treatment experience, the frequencies for 1 = “yes, I sought help” are reported

Comparing the *milieux* to each other, there are significant differences. On average, participants from the *family bound milieu* are younger and participants from the *self-sufficient milieu* are older than in the other *milieux*. Compared with the overall gender distribution, there are more male participants in the *privileged* and *entitled milieux* than is to be expected. Correspondingly, there are more female participants in the *family bound milieu* than is to be expected. Depression severity is less pronounced in the *privileged* and *entitled milieux* than in the *collaborator* and *family bound milieux*.

Concerning the help-seeking process there are significant differences between the *milieux*. Self-identifying the experienced complaints as symptoms of mental illness is most pronounced in the *collaborator milieu*, especially compared with the *privileged*, *entitled*, and *self-sufficient milieux*. Irrespective of help-seeking source, intention to seek help is most expressed in the *entitled milieu*. Overall intention to seek help is most strongly expressed concerning friends, then family and then from a general practitioner. Help-seeking intention is least expressed concerning a spiritual leader. Corresponding with the higher intention to seek help from a general practitioner, participants in the *entitled milieu* show more than expected help-seeking behaviour. Also, based on the overall help-seeking behaviour frequencies, the *entitled* and *family bound milieux* show more than expected help-seeking behaviour from family members, whilst participants from the *self-sufficient milieu* show less than expected help-seeking behaviour from family members. Concerning previous treatment experience, participants from the *privileged milieu* reported the least experience and participants from the *disadvantaged milieu* reported the most treatment experience.

### Logistic regressions for help-seeking behaviour

The results for the multiple logistic regressions, with the outcome variables *help-seeking behaviour from a general practitioner*, a *mental health professional*, a *friend*, and a *family member*, are reported in Table 3. SES was introduced as a control variable in the respective model 1 of the regression analyses to test the added value of the *Self-Milieux* against already established measures in health-related research. Overall, SES was a positive predictor for help-seeking behaviour.

**Table 3:** Adjusted odds ratios of logistic regression models predicting *help-seeking behaviour* for mental health complaints from general practitioner, mental health professional, friends and family members depending on the *Self-Milieu* a participant belongs to in a sample of adults with depressive complaints (*N* = 1125)

	**General practitioner**			**Mental health professional**			**Friend**			**Family member**		
	*aOR*	95% *CI*		*aOR*	95% *CI*		*aOR*	95% *CI*		*aOR*	95% *CI*	
		Lower	Upper		Lower	Upper		Lower	Upper		Lower	Upper
**Control variables**												
Age	1.03***	1.02	1.04	1.01	0.99	1.02	0.97***	0.97	0.98	0.99	0.98	1.00
Gender (men)^a^	1.16	0.85	1.58	1.17	0.78	1.75	2.11***	1.58	2.81	1.50**	1.13	1.99
Depression severity	1.08***	1.04	1.12	1.06**	1.01	1.10	1.05**	1.01	1.08	1.02	0.99	1.05
Socioeconomic status	1.11**	1.02	1.20	1.03	0.93	1.14	1.07*	1.00	1.15	1.11**	1.03	1.20
Pseudo R^2^ (model 1)	*0.06*			*0.01*			*0.11*			*0.02*		
**Self-milieux**^b^												
1: “the privileged”	0.38***	0.22	0.64	0.53	0.26	1.09	1.52	0.93	2.49	0.30***	0.16	0.59
2: “the collaborators”	0.64	0.39	1.06	0.73	0.37	1.43	1.26	0.76	2.06	0.50*	0.28	0.89
3: “the entitled”	1.00			1.00			1.81***	1.15	2.87	0.77	0.47	1.26
4: “the family bound”	0.88	0.51	1.54	0.94	0.47	1.90	2.11***	1.21	3.65	1.00		
5: “the disadvantaged”	1.05	0.61	1.78	0.81	0.40	1.63	2.20***	1.28	3.78	0.51**	0.33	0.77
6: “the self-sufficient”	0.37***	0.23	0.61	0.81	0.44	1.47	1.00			0.21***	0.12	0.37
Pseudo R^2^ (model 2)	*0.09*^ϯ^			*0.02*			*0.13*^ϯ^			*0.08*^ϯ^		

*aOR* = adjusted Odds Ratio; 95% *CI* = 95% confidence interval for *aOR*; *R*^*2*^ = Nagelkerke’s *R*^*2*^*.* a. reference category in brackets; b. reference category is the group with an *aOR* = 1.00; *p < .05, **p < .01, ***p < .001; ϯ. change in R^2^ is significant

Due to the non-significance of the overall statistical test concerning help-seeking from a mental health professional these results cannot be interpreted but are reported in Table 3. The other regressions were significant and are interpretable.

Concerning help-seeking from a GP, participants from the *privileged* and *self-sufficient milieux* are less likely to seek help than participants from the *entitled milieu*.

Concerning help-seeking from a friend, participants from the *entitled*, *family bound*, and *disadvantaged milieux* are more likely to seek help than participants from the *self-sufficient milieu*.

Concerning help-seeking from a family member, participants from the *privileged*, *collaborators*, *disadvantaged*, and *self-sufficient milieux* are less likely to seek help than participants from the *family bound milieu*.

## Discussion

The aim of the present study was to present and test the novel framework, *Self-Milieux*, that represent individuals sociocultural background in an economic way for empirical research. To show the predictive validity the *Self-Milieux* framework was exemplarily tested in the context of the help-seeking process for mental health issues [50].

It is argued that both the social as well as the cultural background are relevant for the development of self-construal, synergistically coming together and representable as *Self-Milieux*. People within the same *milieu* have similar self-understanding and construal but do not represent actual social groups, as is the aim of social milieu concepts [21]. Furthermore, it is argued that the understanding of self will have an impact on help-seeking and non-help-seeking behaviour. This study answers the call to regard and recognise individual health-related behaviour on a psychological as well as sociocultural level [16–19].

As a means of introducing a conceptually consistent methodology to determine the *Self-Milieux* a two-stage clustering analysis method was used [60]. It is a method allowing for re-allocation of cluster membership. Cohens Kappa was moderate, indicating that a sufficient number of participants were allocated to the same cluster in both stages but that some necessary miss-allocations were rectified and individuals reassigned to the nearest cluster centroid. Six clusters were chosen to represent different *milieux* based on the hierarchical cluster dendrograms and explanatory plausibility [62]. The clusters are distributed equally across the three SES status groups which is due to the chosen methodology representing relative status groups [56]. Two of the clusters are mainly *inter*dependent, whereas four are mainly *in*dependent with significant differences concerning the self-construal dimensions. It is important to note that on a conceptual level the horizontal dimension ranges from highly *inter*dependent to highly *in*dependent. The empirical model however shows less extreme self-construal profiles. This is likely due to a study sample bias and the limitation that only people registered at the online panel could participate (see Sect. “Strengths and limitations”).

Also, there is some criticism concerning the method itself. First, the cluster analysis process is inherently conditional and decisionistic, meaning that other cluster solutions would have been possible. Second, it is criticised that there is a lack of standardised reporting guidelines making it difficult to compare and replicate studies [70]. However, because this is an introduction of a novel framework the methodology is considered sufficient and in the supplementary material the R-code was provided so that replication is possible.

To show the predictive validity, the *Self-Milieux* are exemplarily examined regarding variables of the help-seeking process [50]. The main finding is that participants from different *milieux* show significant differences when it comes to the separate parts of this help-seeking process and how these are associated with each other. Importantly, the resulting differences could be shown with the socioeconomic status introduced in the models as a control variable due to its established role in health related behaviour [e.g., 34, 38]. Therefore, it is concluded that introducing the *Self-Construal Theory* [45] in such a *milieux* framework has added value for understanding help-seeking behaviour in a health related context. The impact of the milieux on the help-seeking process for the milieu members themselves is discussed as a tendency. Due to the limitations of the study and the constraints of the concept itself, a causal argumentation is not supported.

Participants from *the privileged milieu* generally self-identify complaints as symptoms of mental illness less often, show less intention to seek help and have less than expected treatment experience. They are also less likely to seek help from a general practitioner or a family member. This is in line with research showing that previous steps of the help-seeking process are important for subsequent steps [13, 50, 71, 72]. Participants of this *privileged milieu* are people with mainly independent self-construal and even though they are well educated and belong to an upper income status group they do not seek help for their complaints. Two milieu-characteristics seem to be important here. First, the male proportion is higher than expected, which could explain the reduced help-seeking intention and behaviour [10]. Second, depression severity is lower than in the other milieux, which on the one hand could be because they are from higher SES groups and on the other hand more *in*dependently construed. *In*dependent self-construal has been shown to buffer depression, mediated by higher self-efficacy [49]. The self-efficacy buffer hypothesis seems to be likely, seeing as the results indicated that participants from the more *in*dependent milieux are in general less depressed than the more *inter*dependent milieux. However, when comparing the *privileged milieu* and the *entitled milieu*, participants from the *entitled milieu* show significantly more intention and help-seeking behaviour than participants from the *privileged milieu*, despite their similar SES and SCS profiles and demographic characteristic. One explanation is found when regarding the self-construal profiles more carefully (Table S5). There is a clear difference between the two *milieux* in the SCS dimensions “making decisions”, “communicating with others”, and “dealing with conflicting interests” in which the *entitled milieu* is more *in*dependent than the *privileged milieu*. Therefore, it is suggested that these SCS dimensions could be especially relevant when it comes to help-seeking intention and behaviour; an *in*dependent expression benefitting help-seeking. The actual behaviour and antecedent decision to seek help must come from the individual themselves, even if “others might not approve” or their behaviour is contrary to “other’s expectations” (wordings taken verbatim from the SCS items 3 and 41, Table S2).

An interesting finding is that participants from the more *in*dependent *milieux*, irrespective of SES, are less likely to self-identify their complaints as symptoms of a mental illness, which is most pronounced in the *inter*dependent *milieux*. Possibly, this could be traced back to the *inter*dependent construal of closely sharing self-related aspects within a familial setting whereby family members could reflect the complaints together with the individual and identify the complaints as symptoms of a mental illness. This seem to be especially pronounced in the *collaborators milieu*, in which participants are mainly *inter*dependently construed and from middle and upper SES compared to the *family bound milieu* where the tendency is also more pronounced than in the mainly *in*dependent *milieux* but in which participants are from lower to middle SES than the *collaborators*. This interpretation holds when comparing help-seeking behaviour from a family member. Here, the more *inter*dependent *milieux* are more likely to seek help than the more *in*dependent *milieux*. Even when comparing the *milieux* with similar SES profiles yet clearly contrary self-construal profiles, participants from the mainly *inter*dependent *milieux* are more likely to seek help from family members. This is a good example for the necessity to not only regard SES as an explanans for differences in health-related behaviour, but also regard the self-construal of the person [41].

However, inconsistent with previous research regarding the help-seeking process [50, 52] the higher self-identifying tendency in the *inter*dependent *milieux* does not support a higher intention to seek help or actual help seeking from a general practitioner. An obvious interpretation is that for participants from the mainly *inter*dependent *milieux* further variables impact the help-seeking process in a stronger way than in the other *milieux*. This discrepancy is especially pronounced when comparing the *collaborators* and *family bound milieux* with the *entitled milieu*, in which participants show less severe depressive symptoms, lower self-identifying tendency and yet higher help-seeking intention and behaviour. This is one definite indicator that it is expedient to regard help-seekers differentially depending on their sociocultural background. For example, individuals from more *in*dependent *milieux* are supported more, when they are helped to identify their complaints as symptoms of mental illness, e.g., through mental health campaigns. On the other hand, individuals from more *inter*dependent *milieux*, are more likely to find support when the peers and family members are shown the necessity and possibility to seek help from an out-group source, e.g., a general practitioner.

### Strengths and limitations

There are some important strengths and limitations. A strength is the adaptability of the cluster methodology and the SES indexing procedure [56], meaning that the proposed methods could be easily used in different social and cultural environments. The novel framework also explains variances in help-seeking behaviour and could be used to develop intervention material aimed specifically for target groups who are less likely to seek professional help despite acknowledging their complaints as symptoms of a mental illness, i.e., more *inter*dependently self-construed individuals. The sample was fitting to exemplarily test the *Self-Milieux* in empirical research, because participants reported symptoms of depression but were not in treatment at the beginning of the study. Due to the longitudinal design, actual help-seeking behaviour in the six-month period could be analysed.

Nevertheless, there are some important limitations. First and foremost, the data is based on self-reports which is inherently biased and does not represent actual help-seeking behaviour. Second, the sample was acquired using an online panel and has to be considered a convenience sample. Although the panel aims to be representative, there is likely a participation-selection bias, e.g., participants with high motivation to participate in online studies, participants with higher levels of education and income (as seen in this sample). Furthermore, it is unlikely that many individuals participated who are from very low SES backgrounds or ethnic minority groups. Therefore, a replication of the study would be interesting with data from a broader and more representative (German) sample as well as amongst hard to reach minority groups. Additionally, the differences between the six milieux might be impacted by the sample composition. Third, the impact of the milieux on the help-seeking process might primarily be attributed to self-construal profiles, as SES was introduced as a control variable in the regression analyses. This was done because SES is an established measure. Nevertheless, a novel framework must demonstrate the ability to add explanatory value to established concepts in order to be accepted. The findings provide support for the idea that the milieux go beyond the original constitutional variables in a Gestalt manner. Fourth, the study was conducted online during the COVID-19 pandemic, from January to September 2021, and even though research shows that online assessment doesn’t reduce attention levels [73] the online assessment will have had an impact on the external validity of the results. However, to counteract this issue, strict data control exclusion criteria were used before data analysis. Lastly, the self-construal items are usually not meant to be averaged into one score [31] which was done here to simplify visualisation (Fig. 1). However, internal consistency across all items is very good and because cluster centroids are reported, interpretation on a dimensional level was still possible.

### Conclusions

This study attempts to combine findings from relative disparate research traditions and combine them into a novel *Self-Milieux* framework that goes beyond the separate determinants to explain health related help-seeking behaviour, both of a formal and an informal source. Participants from some *milieux* show a help-seeking process that has been proposed in previous research [13, 50] while other participants from more *inter*dependent *milieux* show a different process, one that ends in informal help-seeking and is less influenced by self-identifying complaints as symptoms of a mental illness. Furthermore, these findings confirm the necessity to differentiate between similar SES groups when researching health related behaviour [41]. It is our conclusion, therefore, that understanding help-seeking in a *milieux* framework has practical relevance, because it should be useful in developing *milieux*-sensitive strategies to support individuals seeking (mental) health care. Possibly, such a campaign diversifying help-seeking strategies could counteract some troubling societal tendencies in which health related inequalities are becoming more and more prominent despite political and scientific efforts to counteract this inequality.

## Supplementary Information

Below is the link to the electronic supplementary material.

Supplementary file1 (DOCX 88 KB)

## Data Availability

The datasets generated during the current study is available from the corresponding author on reasonable request.
