# Supplementary material for: A “Self-Milieux” perspective on help-seeking: examining the impact of a person’s sociocultural background on help-seeking in people with untreated depressive symptoms
Source: Soc Psychiatry Psychiatr Epidemiol. 2024 Aug 4;60(3):579–92. doi: 10.1007/s00127-024-02720-3 (PMC11870981; doi:10.1007/s00127-024-02720-3)
Supplement: Supplementary file 1 — Supplementary file1 (DOCX 88 KB) [file 127_2024_2720_MOESM1_ESM.docx]

Supplementary Material to the article:

McLaren et al. (2024). **A “Self-Milieux” perspective on help-seeking: examining the impact of a person’s sociocultural background on help-seeking in people with untreated depressive symptoms**. *Social Psychiatry and Psychiatric Epidemiology*

# Content

In this supplement we report the exact calculation basis for the *Socioeconomic Status-Index* (tab. S1) and report the *Self-Construal Scale* (Vignoles et al., 2016) items/dimensions used to determine the self-construal profile (tab. S2). Furthermore, the R script for the two-stage clustering method to determine the “Self-Milieux (tab. S3), the dendrogram showing the hierarchical cluster solutions based on the ‘hclust’ {stats} algorithm (tab. S4), the calculated centroids from the hierarchical cluster method (ward method; tab. S5), as well as the different cluster membership depending on the *agnes* {cluster} or the *kmeans* {stats} cluster algorithm (tab. S6) are reported.

| **Table S1.** Basis for calculating the *Socioeconomic Status Index* based on sociodemographic data assessed during the baseline assessment time. | | |
| --- | --- | --- |
| **"Education Index": schooling qualifications** | **"Vocation Index": vocational status** | **"Income index": net household income** |
| No school qualification *AND* no vocational qualification  **(= 1,0)** | Unemployed *OR* not yet employed (including: pupils, students) **(= 1.0)** | 0-500 € **(= 1)** |
| Lower secondary certificate *AND* no vocational qualification **(= 1,7)** | Marginally employed, 400€ job *OR* "one-euro-job" (when receiving unemployment benefits), *OR* occasionally or irregularly employed, *OR* permanently unemployed  **(= 1.5)** |  |
|  | In vocational training/ apprenticeship *OR* in retraining *OR* military service/civilian service *OR* voluntary social year/ federal volunteer service *OR* pupils at a general school **(= 1,8)** |  |
| Secondary school diploma/-POS *AND* no vocational qualification **(= 2.8)** | Housewife/househusband *OR* maternity leave, parental leave or other leave of absence **(= 2,3)** | 501-1000 € **(= 2)** |
|  | Students **(= 2.8)** |  |
| No school qualification *OR* lower secondary certificate *AND* vocational-in-company training *OR* vocational-school education *OR* education at a technical school, master school, technical school, vocational or technical academy **(= 3.0)** | Students *AND* self-employed **(= 3.2)** | 1001-1500 € **(= 3)** |
| Secondary school diploma/-POS *AND* vocational-in-company training *OR* vocational-school education *OR* education at a technical school, master school, technical school, vocational or technical academy **(= 3.6)** | Retired persons, pensioners, early pensioners **(= 3.4)** |  |
| Advanced technical college entrance qualification, completion of specialised upper secondary school *OR* general university entrance qualification/ A-levels/EOS *AND* no vocational qualification **(= 3.7)** | Partial retirement **(= 3.6)** |  |
| Advanced technical college entrance qualification, completion of specialised upper secondary school *OR* general university entrance qualification/ A-levels/EOS *AND* vocational-occupational training *OR* vocational-school training *OR* education at a technical school, master school, technical school, vocational or technical academy **(= 4.8)** | *ONLY* part-time employed  **(= 4.2)*** | 1501-2000 € **(= 4)** |
|  | *ONLY* self-employed **(= 4.5)*** |  |
|  | Part-time employed *AND* self-employed **(= 4.8)*** |  |
| Advanced technical college entrance qualification, completion of specialised upper secondary school *OR* general university entrance qualification/ A-levels/EOS *AND* advanced technical college degree (Bachelor) *OR* university degree (Bachelor) (**= 5.1)** | Full-time employed *ONLY*  **(= 5.2)*** | 2001-2500 € **(= 5)** |
| Advanced technical college entrance qualification, completion of specialised upper secondary school OR general university entrance qualification/ A-levels/EOS *AND* advanced technical college degree (e.g. Diploma, Master) *OR* university degree (e.g. Diploma, Magister, State examination, Master) **(= 5.8)** |  |  |
| General qualification for university entrance/ A-levels/EOS *AND* doctorate  **(= 6)** | Full-time employed *AND* self-employed **(= 6.0)*** | 2501 € or more **(= 6)** |
| **Notes.** The logical term *OR* means exclusive “either, or”, because only one answer concerning this variable could be given. The logical term *AND* means that both one of the answers before and after the AND had to be given by the participant.  * If full-time, part-time *OR* self-employed *AND* an indication from the categories "1" *OR* "2", then the mean score is given, e.g., full-time *AND* part-time, 400-euro job, mini-job equals an index value of 3.35 = (5.2 + 1.5) / 2  In brackets, the index values allocated to the individual participants if they met the necessary conditions.  Some answers will not apply to international samples (e.g., *POS*, which is a *polytechnic secondary school qualification*) but are supplied here, as they are part of the answers participants could state during the survey in the project implemented in a German sample. | | |

| **Table S2.**  In this table the *Self-Construal Scale* items are shown, separately for each dimension |
| --- |
| **Making decisions: Self-direction versus reception to influence** |
| Item 3: You always make your own decisions about important matters, even if others might not approve of what you decide. |
| Item 28: You decide for yourself what goals to pursue even if they are very different from what your family would expect. |
| Item 41: You usually decide on your own actions, rather than follow others’ expectations. |
| *Item 7: You usually ask your family for approval before making a decision.* |
| *Item 19: You usually follow others’ advice when making important choices.* |
| *Item 33: You usually do what people expect of you, rather than decide for yourself what to do.* |
| **Looking after oneself: Self-reliance versus dependence on others** |
| Item 9: You tend to rely on yourself rather than seeking help from others. |
| Item 34: You prefer to rely completely on yourself rather than depend on others. |
| Item 45: You try to avoid being reliant on others. |
| *Item 14: In difficult situations, you tend to seek help from others rather than relying only on yourself.* |
| *Item 29: Being able to depend on others is very important to you.* |
| *Item 43: You prefer to ask other people for help rather than rely only on yourself.* |
| **Communicating with others: Self-expression versus harmony** |
| Item 4: You show your true feelings even if it disturbs the harmony in your family relationships. |
| Item 35: You prefer to express your thoughts and feelings openly, even if it may sometimes cause conflict. |
| Item 46: You like to discuss your own ideas, even if it might sometimes upset the people around you. |
| *Item 10: You prefer to preserve harmony in your relationships, even if this means not expressing your true feelings.* |
| *Item 20: You try to adapt to people around you, even if it means hiding your feelings.* |
| *Item 44: You try not to express disagreement with members of your family.* |
| **Dealing with conflicting interests: Self-interest versus commitment to others** |
| Item 11: You usually give priority to your personal goals, before thinking about the goals of others. |
| Item 21: Your own success is very important to you, even if it disrupts your friendships. |
| Item 30: You protect your own interests, even if it might sometimes disrupt your family relationships. |
| *Item 25: You value good relations with the people close to you more than your personal achievements.* |
| *Item 36: You usually give priority to others, before yourself.* |
| *Item 47: You would sacrifice your personal interests for the benefit of your family* |
| **Note.** Items measuring independent self-construal in normal script and items measuring interdependent self-construal in *italic* script; item ratings range from 1 = “doesn’t describe me at all” to 5 = “describes me exactly”; item numbers refer to the original scale “Culture and Identity Research Network Self Construal Scale Version 3 (CIRN-SCS-3)” by Vignoles et al. (2016) |

| **Table S3.**  R script for the two-stage clustering method (Milligan & Sokol, 1980) to determine the “Self-Milieux”. |
| --- |
| **#packages####**  #install.packages("stats") # for the functions hclust and kmeans  #install.packages("cluster") # for the functions agnes  #install.packages("DescTools") # to compute Cohens Kappa as a means to compare cluster solutions  **#hierarchical clustering_agnes####**  ## using the "cluster" package, "agnes" function  *library(cluster)*  ## many of the following arguments are at “default”, though for scientific transparency (especially for R-software lay people) we opted to explicate our  ## script to show exactly what was done  *analysis_agnes <- agnes(data[,c('ZSES_Bildung', 'ZSES_Beruf', 'ZSES_Einkommen_household', 'Zmaking_decisions','Zlooking_after_oneself',*  *'Zcommunicating_with_others', 'Zdealing_with_conflicting_interests')],*  *metric = "euclidean", diss = FALSE, keep.data = FALSE,*  *stand = FALSE, method = "ward")*  ## ploting a dendogram of an 'agnes' object  *plot(analysis_agnes, ask = FALSE, which.plots = 2, main = NULL, sub = paste("Agglomerative Coefficient = ",round(analysis_agnes$ac, digits = 2)),*  *adj = 0, nmax.lab = 35, max.strlen = 5, xax.pretty = TRUE)*  ## add clustering solution (cutree) to the data  *clusters_agnes <- cutree(analysis_agnes, k = 6) # cluster solution based on the dendogram, throretical/intuitive reasoning*  *data <- cbind(data, clusters_agnes)*  **#hierarchical clustering_hclust####**  ## distance matrix, the distance between every point in your data frame to every other point  *distance_matrix <- dist(data[,c('ZSES_Bildung', 'ZSES_Beruf', 'ZSES_Einkommen_household', 'Zmaking_decisions','Zlooking_after_oneself',*  *'Zcommunicating_with_others', 'Zdealing_with_conflicting_interests')],*  *method = "euclidean")*  ## fitting function, "ward.D2" is the method as argued by Ward  *analysis_hclust <- hclust(distance_matrix, "ward.D2")* # same as "ward" method in agnes (Murtagh & Legendre)  ## dendogram, to decide where to "cut" the cluster tree and merge data into clusters  *plot(analysis_hclust)*  *rect.hclust(analysis_hclust, k = 6, border = "blue")* # to visualize the cutting  ## add clustering solution (cutree)to the data  *clusters_hclust <- cutree(analysis_hclust, k = 6)*  *data <- cbind(data, clusters_hclust)*  **#determining the cetroids for use in the non-hierarchical method####**  ## You can use the clustering to assign cluster membership and then calculate the centre for all the observations in a cluster.  ## The k-means function allows you to specify initial centres via the centroids  *centroids = aggregate(data[,c('ZSES_Bildung', 'ZSES_Beruf', 'ZSES_Einkommen_household',*  *'Zmaking_decisions','Zlooking_after_oneself', 'Zcommunicating_with_others', 'Zdealing_with_conflicting_interests')],*  *list(cluster=clusters_agnes),mean)*  **#non-hierarchical clustering_kmeans####**  ## clustering using the k-means function, with pre-defined centroids  *analysis_kmeans <- kmeans(data[,c('ZSES_Bildung', 'ZSES_Beruf', 'ZSES_Einkommen_household', 'Zmaking_decisions','Zlooking_after_oneself',*  *'Zcommunicating_with_others', 'Zdealing_with_conflicting_interests')],*  *centroids[,-1],*  *iter.max = 25, nstart = 1,*  *algorithm = "MacQueen",trace = FALSE)*  ## add clustering solution to the data  *clusters_kmeans <- analysis_kmeans[["cluster"]]*  *data <- cbind(data, clusters_kmeans)*  **#Cohens kappa to compare the cluster solutions####**  *library(DescTools)*  ## check to see if the hierarchical cluster solutions have similar or same results  *cluster_solutions_1 <- xtabs (~ clusters_agnes + clusters_hclust)*  *cluster_solutions_1*  *CohenKappa(cluster_solutions_1, conf.level = 0.95) #if the solutions are the same, then Kappa should be 1*  ## comparing the cluster solutions when using the centroids determined by the hierarchical method  *cluster_solutions_2 <- xtabs (~ clusters_agnes + clusters_kmeans)*  *cluster_solutions_2*  *CohenKappa(cluster_solutions_2, conf.level = 0.95)*  #Kappa value interpretation Landis & Koch (1977): <0 No agreement; 0 - .20 Slight; .21 - .40 Fair; .41 - .60 Moderate; .61 - .80 Substantial; .81-1.0 Perfect |

**Figure S4**

Dendrogram showing the hierarchical cluster solutions based on the ‘hclust’ {stats} algorithm. The rectangles show the chosen cluster solution with a cut-off Hight of 25 (k = 6).


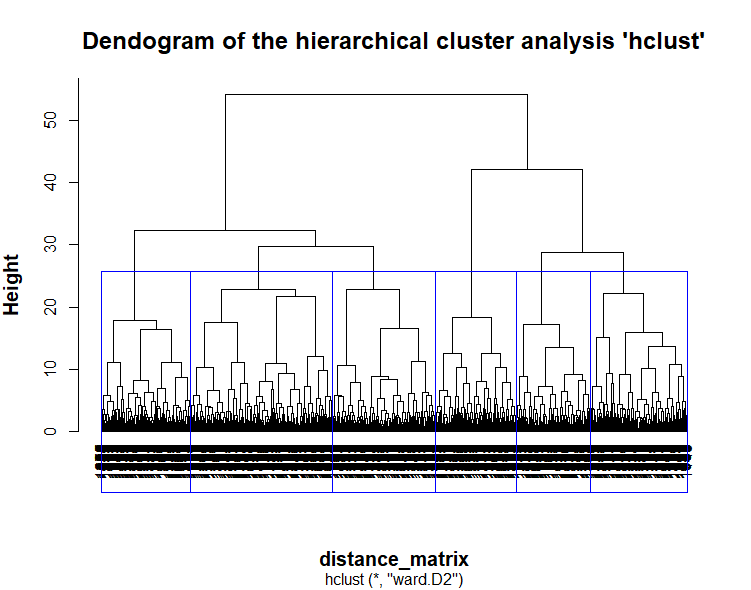


| **Table S5.**  Centroids for the six clusters chosen as the best solution | | | | | | | |
| --- | --- | --- | --- | --- | --- | --- | --- |
|  | **Socioeconomic status** | | | **Self construal profile** | | | |
|  | **education** | **vocation** | **income** | **making decisions** | **looking after oneself** | **communica-ting with others** | **dealing with conflicting interests** |
| 1 | 0.9618 | 0.7812 | 1.1662 | -0.2237 | -0.2185 | 0.0236 | 0.1256 |
| 2 | -0.4224 | 0.4764 | -0.0240 | -0.8739 | -0.0419 | -0.7710 | -0.8027 |
| 3 | 0.0545 | 0.3957 | 0.0214 | 0.1227 | -0.2440 | 0.6097 | 0.4045 |
| 4 | -0.4808 | -0.9946 | -1.0332 | -0.7757 | -0.5734 | -0.4278 | -0.4111 |
| 5 | -0.4047 | -1.3197 | -0.7240 | 0.7942 | 0.4002 | 0.5022 | 0.3777 |
| 6 | 0.0426 | 0.3513 | 0.4194 | 0.9139 | 0.9386 | -0.3496 | -0.0184 |
| **Note.** The values are given in their z-standardised forms and, therefore, cannot be interpreted exactly on the respective scales. However, interpretation of tendencies from high (+) to low *socioeconomic status* (-) and *in*dependent (+) to *inter*dependent *self-construal* (-) is possible. | | | | | | | |

| **Table S6.**  Cross table of cluster membership depending on the *agnes* {cluster} or the *kmeans* {stats} cluster algorithm which is done with the centroids of the *agnes* solution in a two-stage method | | | | | | |
| --- | --- | --- | --- | --- | --- | --- |
| **agnes** | **kmeans** | | | | | |
|  | 1 | 2 | 3 | 4 | 5 | 6 |
| 1 | **157** | 59 | 39 | 2 |  | 13 |
| 2 |  | **112** | 18 | 33 | 1 | 30 |
| 3 | 80 | 25 | **180** | 19 | 41 | 27 |
| 4 |  | 10 | 20 | **190** | 28 | 6 |
| 5 | 1 |  | 6 | 22 | **179** | 4 |
| 6 | 47 | 2 | 5 | 1 | 15 | **163** |
| **Note.** There are a number of cross cluster membership allocations, depending on whether a hierarchical method (*agnes*) or a non-hierarchical method (*kmeans*) is used. No numeric value in the cell equals 0. Cohens Kappa is moderate with 0.57 [95% CI: 0.54; 0.59] | | | | | | |

References

Milligan, G. W., & Sokol, L. M. (1980). A Two-Stage Clustering Algorithm with Robust Recovery Characteristics. *Educational and Psychological Measurement*(40), 755–759.

Murtagh, F., & Legendre, P. Ward’s hierarchical agglomerative clustering method: which algorithms implement Ward’s criterion? *Journal of Classification*, *2014*(31(3)), 274–295.

Vignoles, V. L., Owe, E., Becker, M., Smith, P. B., Easterbrook, M. J., Brown, R., Gonzalez, R., Didier, N., Carrasco, D., Cadena, M. P., Lay, S., Schwartz, S. J., Des Rosiers, S. E., Villamar, J. A., Gavreliuc, A., Zinkeng, M., Kreuzbauer, R., Baguma, P., Martin, M., . . . Bond, M. H. (2016). Beyond the 'east-west' dichotomy: Global variation in cultural models of selfhood. *Journal of Experimental Psychology: General*, 966–1000. https://doi.org/10.1037/xge0000175
